# Identification of a Ferroptosis Gene Set That Mediates the Prognosis of Squamous Cell Carcinoma of the Head and Neck

**DOI:** 10.3389/fgene.2021.698040

**Published:** 2021-09-03

**Authors:** Chunyan Li, Xuemin Wang, Rujia Qin, Zhaoming Zhong, Chuanzheng Sun

**Affiliations:** ^1^Department of Head and Neck Surgery Section II, The Third Affiliated Hospital of Kunming Medical University/Yunnan Cancer Hospital, Kunming, China; ^2^Department of Medical Oncology, The First Affiliated Hospital of Kunming Medical University, Kunming, China

**Keywords:** HNSCC, ferroptosis-related genes, worse prognosis, immune cell infiltration, DEGs

## Abstract

Squamous cell carcinoma of the head and neck (HNSCC) is one of the six most common malignancies. HNSCC has both a high incidence and poor prognosis, and its prognostic factors remain unclear. Ferroptosis is a newly discovered form of programmed cell death that is iron-dependent. Increasing evidence indicates that targeting ferroptosis may present a new form of anti-tumor treatment. However, the prognostic value of ferroptosis-related genes (FRGs) in HNSCC is unclear. This study was designed to identify molecular markers associated with ferroptosis that influence prognosis in patients with HNSCC. We used HNSCC tumor and normal data from The Cancer Genome Atlas (TCGA) to identify prognosis-related FRGs. An FRG-based prognostic risk score was constructed, and its prognostic value for patients with HNSCC was evaluated using receiver operating characteristic curve (ROC) and nomogram analyses. The model was validated using the Gene Expression Omnibus (GEO) database. Univariate Cox regression analysis in patients with HNSCC revealed 11 FRGs that were significantly associated with overall survival (OS). We constructed a ferroptosis risk score model based on five genes and divided the patients into different risk groups based on its median value. Kaplan-Meier curve analysis showed that patients with a higher ferroptosis risk score had shorter OS (TCGA training set: *P* < 0.001, TCGA validation set: *P* < 0.05,GEO validation set: *P* < 0.001), and Gene Expression Profiling Interactive Analysis (GEPIA) further verified the relationships between these five genes and prognosis in patients with HNSCC. Multivariate Cox regression analysis showed that the risk score remained an independent predictor of OS after the exclusion of clinical confounders (*HR* > 1, *P* < 0.01). Significant differences in gene function enrichment analysis and immune cell infiltration status were identified between the two groups. The prognostic model can be used to predict the prognosis of patients with HNSCC. Moreover, the five FRGs may affect ferroptosis in HNSCC and thereby represent potential treatment targets. These results provide new directions for HNSCC treatment.

## Introduction

Squamous cell carcinoma is a common malignant tumor of the head and neck (HNSCC). The incidence of HNSCC is trending upwards, with approximately 600,000 new cases diagnosed yearly (Torre et al., 2015; Shield et al., 2017; Solomon et al., 2018). In the past 40 years, despite continuous advances in radiotherapy equipment and surgical techniques, the 5-year survival rate of patients with HNSCC has remained at approximately 50%, largely due to its high recurrence rate and the disability rate associated with radiotherapy and surgical treatment (Koyfman et al., 2019). Clinical studies have shown that adjuvant therapy for advanced HNSCC using platinum-based chemotherapy is effective and can significantly improve patient survival rates. However, because some patients are insensitive to chemotherapy or develop drug resistance after treatment, the overall 5-year survival rate associated with chemotherapy remains low (Vidal et al., 2017; Wang et al., 2020). The only molecular targeted therapy that is approved for the treatment of HNSCC is cetuximab, but its clinical response rate is modest (only 10–15%) (Vermorken et al., 2007; Hammerman et al., 2015; Mandal et al., 2016). The complex mutation landscape of HNSCC may be the main reason for the low response rate of targeted therapy, as most tumors are caused by multiple genetic drivers that have evolved further after multiple rounds of chemoradiotherapy. This highlights the importance of targeted therapy selection for tumors. Iron-dependent cell death, called ferroptosis, had been identified as a form of programmed cell death. A growing number of studies suggest that ferroptosis may be a new cancer therapy strategy, potentially avoiding the need to target complex and redundant molecular pathways (Latunde-Dada, 2017; Yu et al., 2017; Hirschhorn and Stockwell, 2019). In addition to the traditional ferroptosis inducer erastin (Sun et al., 2016), sorafenib also induces ferroptosis. Ferroptosis also participates in the CD8 + T cell-mediated antitumor effects induced by immunotherapy (Wang et al., 2019). In addition, some cancer-related genes, including *TP53* and *ALOX12*, may be important ferroptosis drivers (Jiang et al., 2015; Chu et al., 2019). In contrast, *GPX4* and *FSP1* negatively regulate ferroptosis (Imai et al., 2017; Bersuker et al., 2019; Doll et al., 2019; Seibt et al., 2019). Numerous studies note the significant enrichment of ferroptosis-related genes (FRGs) in HNSCC, suggesting that ferroptosis targeting may be a new approach for treating advanced HNSCC (Ye et al., 2019; Liu et al., 2020; Yu et al., 2020). However, further advances in ferroptosis-targeting strategies for HNSCC require a detailed understanding of FRG expression patterns in different types of HNSCC. We analyzed The Cancer Genome Atlas (TCGA) database transcriptome data of 506 tumors to comprehensively describe differences in FRG transcription levels in HNSCC, and to provide a theoretical basis for ferroptosis-targeting HNSCC treatments. The objective of this study was to analyze FRG transcriptional variations in HNSCC to elucidate FRG expression profiles and assess the association between FRGs and clinical prognosis. Increasing evidence indicates that ferroptosis is also involved in immunotherapy. We therefore compared the immune infiltration status of different ferroptosis risk groups. Understanding the level of ferroptosis in HNSCC may help to develop a theoretical basis for ongoing basic research in ferroptosis oncology, accelerate the development of therapeutic ferroptosis targets in HNSCC, and guide clinical research.

## Materials and Methods

### Collection and Processing of TCGA Cohort and GEO Cohort Data

Gene expression data of RNAseq counts of 546 patients with HNSCC were downloaded from UCSC Xena. Related clinical data and survival data were also retrieved from the UCSC Xena (Table 1). Gene expression data were normalized using the variance stabilization transformation method of the “DEseq2” R software package. Gene expression annotation information was downloaded from the Ensembl website.^1^ As the downloaded data was logarithmic in value, it needed to be restored to its original count value prior to comparative analysis. We used the “Deseq2” R software package to analyze the genetic difference between HNSCC tumor tissues and normal tissues to identify differentially expressed genes (DEGs). RNAseq data of 97 additional tumor samples and clinical information was download from the Gene Expression Omnibus (GEO) database^2^ and analyzed with the “limma” package for internal standardization. Gene sequencing data annotation information was downloaded from the Bioconductor with the R package “hgu133plus2 GPL570 platform.”

**TABLE 1:** Clinical characteristics of patients with squamous cell carcinoma of the head and neck (HNSCC) enrolled in this study.

		TCGA training set (326)			TCGA validation set (140)			GEO validation set (97)		
				
		High risk	Low risk	*p* value	High risk	Low risk	*p* value	High risk	Low risk	*p* value
**Age(years)**				0.073			0.734			0.84
	<60	62	79		33	30		26	24	
	≥60	101	84		37	40		23	24	
**Gender**				0.533			0.569			0.664
	Male	116	122		53	49		32	34	
	Female	47	41		17	21		17	14	
**T stage**				0.49			0.001			
	T1-2	56	63		14	33		NA	NA	
	T3-4	107	100		56	37		NA	NA	
**lymph node metastasis**				0.438			0.865			
	Yes	74	82		39	37		NA	NA	
	No	89	81		31	33		NA	NA	
**Metastasis**				0.562			0.12			
	Yes	2	1		3	0		NA	NA	
	No	161	162		67	71		NA	NA	
**Stage**							0.127			0.066
	I-II	43	39		9	17		16	25	
	III-IV	120	124		61	53		23		

### Identification of FRGs and Immune Cell Infiltration Status

A table of FRGs, containing six datasets, was obtained from the FerrDb web portal.^3^ We analyzed drivers, suppressors, and markers of ferroptosis in this study, and 259 genes were considered for further experiments (Supplementary Table 1).

Single-sample gene set enrichment analysis (ssGSEA) was used to evaluate the immune infiltration status of patients in different risk groups (Supplementary Table 2).

### Construction of a Prognostic Model Containing FRGs and Verification of Its Predictive Ability

Differentially expressed genes (DEGs) between HNSCC and normal tissues were identified with the “Deseq2” R package in the TCGA cohort (padj < 0.05 and|log2FC| ≥ 1). Online software^4^ and the “heatmap” R package were used to generate the Venn diagram and heatmap, respectively. The STRING online database was used for protein interaction network analysis, which was visualized with Cytoscape (version 3.7.1). Most plots in this study were drawn using ggplot2.

To investigate the association of clinical pathologic characteristics and FRGs with overall survival (OS) in HNSCC, we randomly divided 466 patients with complete clinical information from TCGA into a training set (*N* = 326) and a validation set (*N* = 140) using a ratio of 7:3. In the training set, univariate Cox regression analysis was used to identify FRGs significantly related to prognosis, and *P*-values were adjusted with Benjamini and Hochberg analysis. To further identify those FRGs that were independent prognostic factors for HNSCC patients, a multivariate Cox regression analysis was used to exclude the effect of clinical confounders. We evaluated the impact of each factor on prognosis by calculating the hazard ratio (HR) and 95% confidence interval (CI). To further investigate the effect of differential FRGs on HNSCC prognosis, we constructed a risk score model including five FRGs. The risk score was calculated as risk score = Σ (coefi × expi). The samples in the training set, validation set, and GEO validation set were divided into two different risk groups based on the median value of the risk score. Receiver operating characteristic (ROC) curve and nomogram analyses were used to assess the ability of the risk score to predict prognosis. Correlation analysis was performed using Pearson tests. A Kaplan-Meier curve for OS was generated, and the log-rank test was used to identify differences in OS between different risk groups. The “prcomp” function of the “stats” R package was used for principal component analysis (PCA). The Gene Expression Profiling Interactive Analysis (GEPIA) online database^5^ was used to further validate differences in gene expression between cancer and normal tissues and the gene expression correlations with prognosis.

### Enrichment Analysis of DEGs

Based on the DEGs (identified with criteria padj < 0.05 and |log2FC| ≥ 1) identified from different risk groups, the R package of “clusterProfiler” was used to perform gene ontology (GO) and Kyoto encyclopedia of genes and genomes (KEGG) analyses. Differences in 16 immune cells and 13 immune-related pathways were analyzed in different risk groups using ssGSEA and the “gsva” R package.

### Nomogram Construction and Evaluation

Based on the coefficients identified in the prognostic model, we developed a nomogram using the “rms” R package. The accuracy of the model prediction was determined by observing the difference between the observed nomogram and the ideal nomogram. The closer the observed nomogram was to the ideal nomogram, the higher the model prediction accuracy.

### qRT-PCR

To further verify the expression levels of five signature genes in HNSCC tumor and normal tissues, we collected tumor tissues from patients with HNSCC who underwent oral squamous cell carcinoma (OSCC) resection at our hospital from January 2020 to March 2021. This study has been reviewed and approved by the Internal Review Committee of our hospital. Total RNA was extracted from HNSCC specimens and normal tissues using the RNA-Quick Purification kit (EScience, Shanghai, China). HiScript^®^ II Q RT SuperMix (Vazyme) was used to synthesize complementary DNA (cDNA). The ChamQ^TM^ SYBR^®^ qPCR Master Mix kit (Vazyme) was used to detect mRNA levels of targeted genes. The human gene primers used were (5′-3′):

GAPDH F-primer: CTCCTGCACCACCAACTGCT,

GAPDH R-primer: GGGCCATCCACAGTCTTCTG;

SLC7A11 F-primer: TCTCCAAAGGAGGTTACCTGC,

SLC7A11 R-primer: AGACTCCCCTCAGTAAAGTGAC;

TRIB3 F-primer: TACCTGCAAGGTGTACCCC,

TRIB3 R-primer: GGTCCGAGTGAAAAAGGCGTA;

AURKA F-primer: GAGGTCCAAAACGTGTTCTCG,

AURKA R-primer: ACAGGATGAGGTACACTGGTTG;

CAV1 F-primer: GCGACCCTAAACACCTCAAC,

CAV1 R-primer: ATGCCGTCAAAACTGTGTGTC;

AKR1C3 F-primer: GTCATCCGTATTTCAACCGGAG,

AKR1C3 R-primer: CCACCCATCGTTTGTCTCGTT.

GAPDH was used to calibrate the relative expression levels of target genes. The primers were synthesized by Synbio Technologies (Suzhou, China). 2^–ΔΔCt^ was used to evaluate relative target gene expression.

### Immunohistochemistry

Immunohistochemistry (IHC) staining was performed using 3.5 μm sections. IHC was used to detect protein expression using AKR1C3 (1:100, A13568, Abclonal), CAV1 (1:100, A1555, Abclonal), AURKA (1:200, bs2749R, bioss), and TRIB3 (1:200, bs7538R, bioss) antibodies. Sections were dewaxed in xylene and rehydrated with graded concentrations of ethanol. Antigen retrieval was performed with sodium citrate antigen for 1.5 min and 3% hydrogen peroxide methanol solution for 30 min to inactivate endogenous horseradish peroxidase. Sections were then incubated with rabbit anti-human AKR1C3, CAV1, TRIB3, and AURKA antibodies. Sections were visualized using the Envision Detection System (Dako) and hematoxylin was used to stain the nucleus.

### Statistical Analysis

Statistical analysis of qRT-PCR results was performed using Prism GraphPad software V8.0 (LaJolla, CA, United States). Gene expression differences between the two groups were calculated with Student’s *t*-test. Correlations between the expression of AKR1C3, CAV1, AURKA, and TRIB3 in HNSCC tissues and clinicopathologic parameters were measured with the chi-square test of SPSS version 23.0. The criterion for determining significant differences was *P* < 0.05. Survival differences between different risk groups were assessed with Log-rank test. Univariate and Multivariate Cox regression analyses were used to evaluate the relationship between clinical factors and patients’ OS. The ssGSEA score for immune cell infiltration status was evaluated in the two different risk groups by Mann-Whitney test. All statistical data analyses were performed using R software (version 4.0.3).

## Results

**FIGURE 1:**
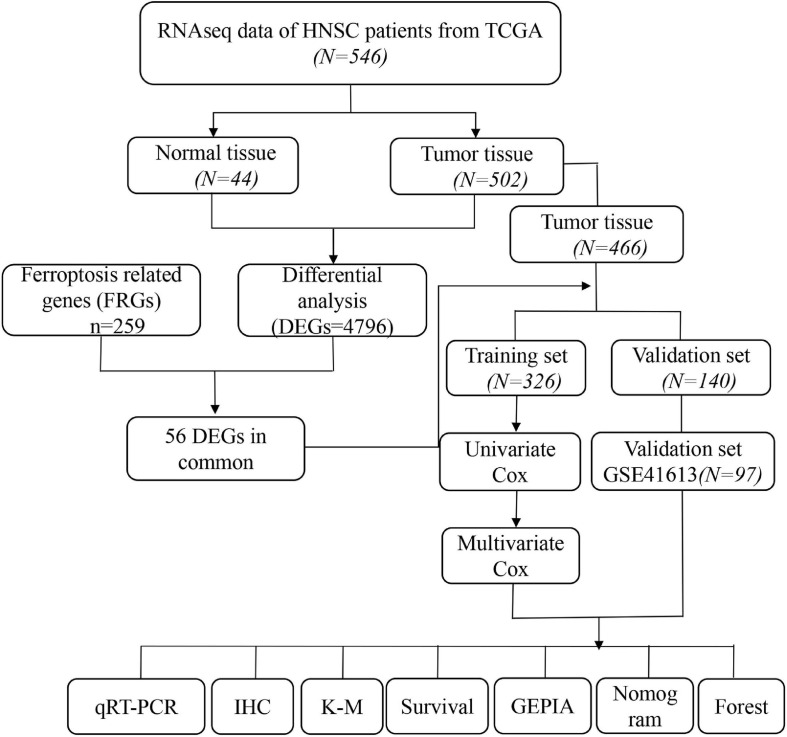
Flow chart of overall study design and analysis.

The overall study design is shown in Figure 1. RNAseq expression data from 466 patients with HNSCC was obtained from the TCGA database and 97 OSCC patients from GEO (GSE41613) were ultimately analyzed. The specific clinical characteristics of the patients are shown in Table 1.

### Identification of DEGs and Prognosis-Related FRGs in the TCGA Cohort

**FIGURE 2:**
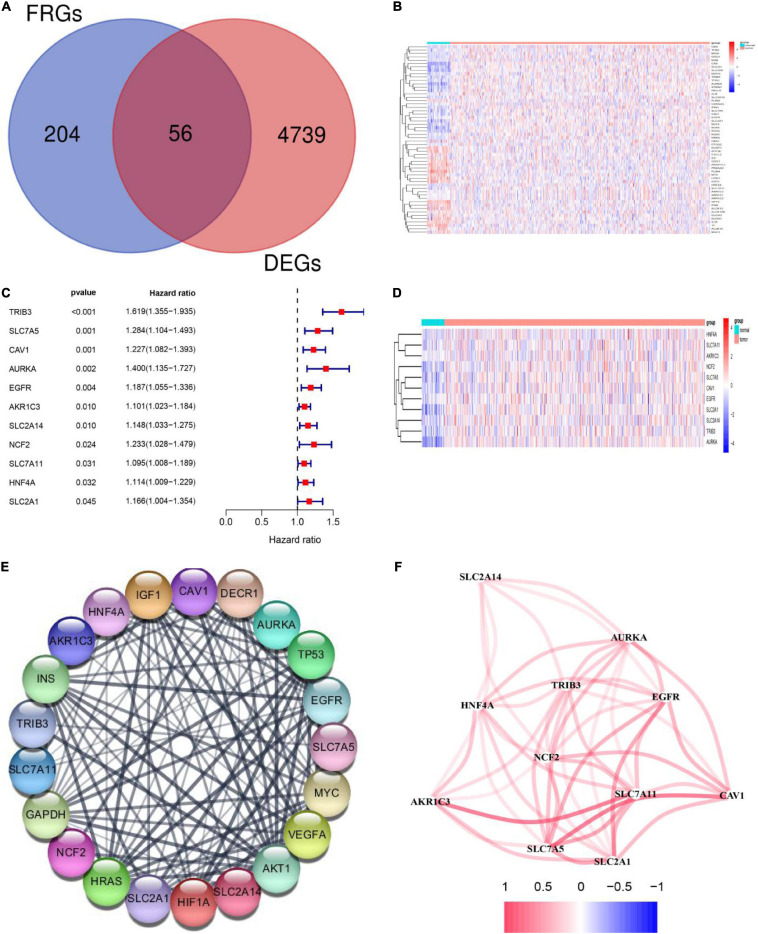
Identification of differentially expressed genes (DEGs) and prognosis-related ferroptosis-related genes (FRGs) in squamous cell carcinoma of the head and neck (HNSCC) using the cancer genome atlas (TCGA) cohort. **(A)** Venn diagram showing DEGs related to ferroptosis in the TCGA-HNSCC cohort. **(B)** The 56 overlapping genes are shown in the heatmap. There were 33 genes that were upregulated and 23 genes that were downregulated in tumor tissues compared with the adjacent normal tissues. **(C)** Univariate Cox regression analysis identified 11 prognosis-related FRGs. **(D)** All 11 FRGs related to prognosis were upregulated in tumor tissues. **(E)** The protein -protein interaction (PPI) network shows interactions between the 11 FRGs related to prognosis. **(F)** The networks related to the 11 FRGs. Different colors represent different correlation coefficients.

RNA expression data was downloaded from TCGA and included 502 HNSCC tumor samples and 44 normal samples. Gene expression values in HNSCC tumor and normal tissues were compared using the “DEseq2” R package. We obtained 4,795 DEGs with the criteria of |log2FC ≥ 1 and padj < 0.05. A series of FRGs (56/259, 21.6%) were significantly differentially expressed between tumor and normal tissues (Figures 2A,B). To further identify the roles of these FRGs in HNSCC, univariable Cox regression analysis was used to identify prognosis-related genes and string was used to perform protein interaction network analysis (Figures 2C–E). We then used the “igraph” R package to construct a core network to identify critical modules and to further screen hub genes. The results indicated that *EGFR*, *SLC2A1*, *SLC7A5*, *SLC7A11*, *AKR1C3*, and *CAV1* are hub genes (Figure 2F).

### Construction of a Prognostic Model Including FRGs With the TCGA Training Set

**FIGURE 3:**
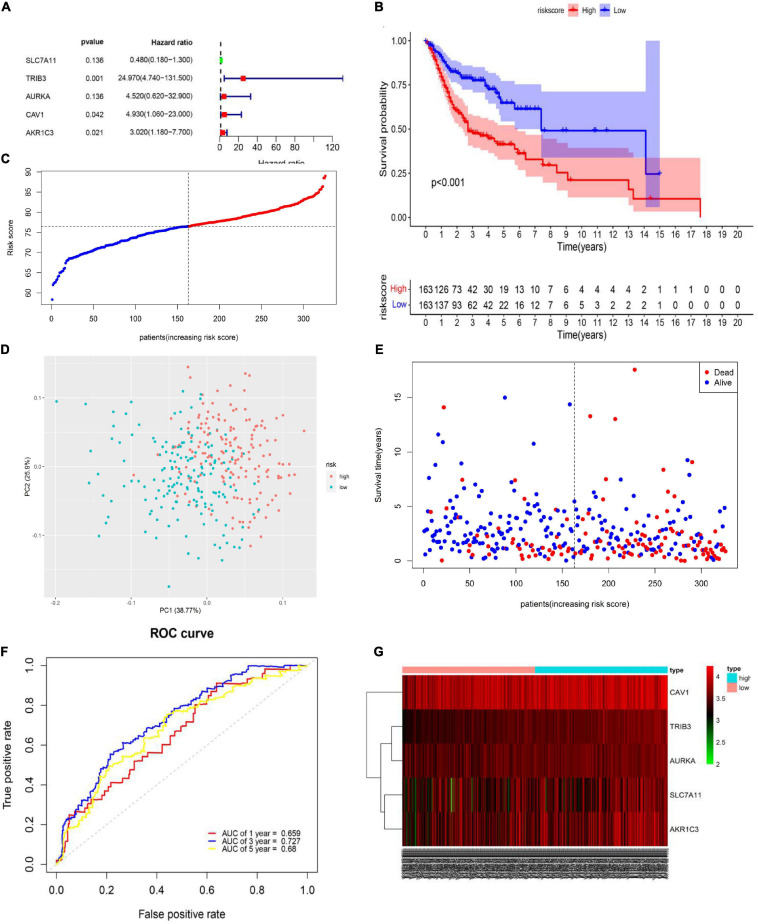
Generation of the prognostic model including ferroptosis-related genes (FRGs) from the cancer genome atlas (TCGA) training set. **(A)** Multivariate Cox regression analysis results. **(B)** Kaplan-Meier curves for the different risk groups in the TCGA training set. Log-rank test, *P* < 0.001. **(C)** Distribution of different risk groups. **(D)** Principal component analysis plot of the TCGA training set. **(E)** Patient survival status and risk score distribution in the TCGA training set. **(F)** Area under the time-dependent receiver operating characteristic curve in the TCGA training set. **(G)** Relationships between prognostic FRG expression levels and different risk groups.

To further explore prognosis-associated FRGs and ferroptosis-related therapeutic targets in HNSCC, we downloaded 466 HNSCC samples with complete clinical information and mRNA expression data from the TCGA database. We then randomly divided the 466 samples into training (*n* = 326) and validation (*n* = 140) sets. Survival analysis using Kaplan-Meier and Univariate Cox regression analyses revealed that the expression levels of the 11 DEGs were significantly related to prognosis in the TCGA training set (Figure 2C). Subsequently, stepwise multivariate Cox regression analysis was used to identify the optimal genes for predicting prognosis. Finally, based on the median value, we constructed a prognostic risk score model containing five genes (*SLC7A11*, *TRIB3*, *AURKA*, *CAV1*, and *AKR1C3*) (Figure 3A). The model indicated that patients with lower risk scores had better prognosis than those with a higher risk scores (Figure 3E). Risk scores were calculated as follows:

Risk score = (−0.73187 ×SLC7A11 expression)+(3.21785 × TRIB3 expression)+(1.5092 × AURKA expression)+(1.59502 × CAV1 expression)+(1.10362 × AKR1C3 expression).

Based on the median value of the risk score (Figure 3C), the TCGA training set was divided into high- (*n* = 163) and low-risk (*n* = 163) groups. Patients in the high-risk group had a shorter OS (high-risk group: 3-year survival rate: 0.4762, 95% CI: 0.3942–0.575; 5-year survival rate: 0.3953, 95% CI: 0.3075–0.508; low-risk group: 3-year survival rate: 0.768, 95% CI: 0.698–0.844, 5-year survival rate: 0.661, 95% CI: 0.561–0.779, *P* < 0.001) than patients in the low-risk group (Figure 3B). PCA showed that patients in the different risk groups were distributed into two clear clusters (Figure 3D). Survival analysis indicated that a higher risk score was associated with higher mortality than a lower risk score. To evaluate the predictive efficacy of our risk prediction model, we performed time-dependent ROC curve analysis and found that the AUC values were 0.659, 0.727, and 0.68 at 1, 3, and 5 years, respectively (Figure 3F). Heatmap analysis showed that high-risk group patients had higher *TRIB3*, *CAV1*, *SLC7A11*, *AURKA*, and *AKR1C3* expression levels than did low-risk group patients (Figure 3G). Detailed information about coefficient profile is summarized in Supplementary Table 3.

### Risk Model Validation Using the TCGA Validation Set and the GEO Validation Set

**FIGURE 4:**
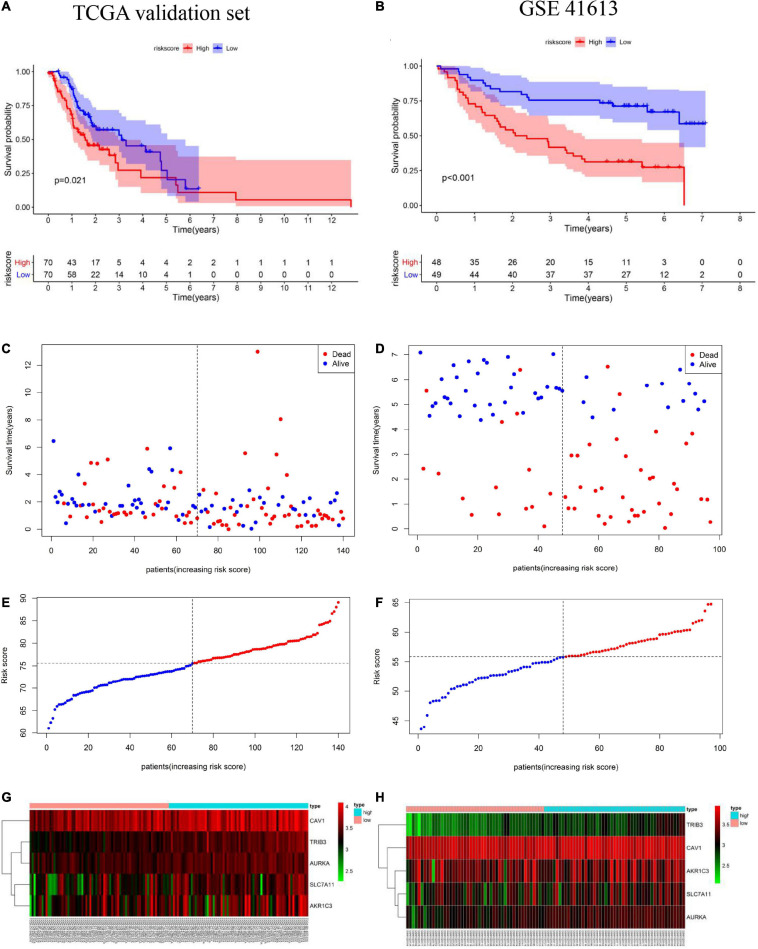
Validation of the prognostic model including ferroptosis-related genes (FRGs) using the cancer genome atlas (TCGA) validation set based on the five gene signature risk scores. **(A,B)** Kaplan-Meier curves for the overall survival of high- and low-risk patients in the TCGA validation set and Gene Expression Omnibus (GEO) validation set. **(C,D)** Relationships between the expression levels of prognostic FRGs and the risk groups. **(E,F)** Distribution of the risk score and the median risk score in the TCGA and GEO cohorts. **(G,H)** Relationship between the expression levels of prognostic FRGs in the different risk groups.

To assess the robustness of the FRG prognostic model, model performance was assessed in the TCGA validation set and in the GEO validation set. A total of 237 patients were used for model validation. For the TCGA validation set 140 patients were divided equally into two different risk groups (Figure 4E). A similar analysis showed that, in the TCGA validation set, patients in the high-risk group had a significantly shorter OS time than those in the low-risk group (Figure 4A). Additionally, higher risk scores were associated with a worse prognosis (Figure 4C). Heatmap results showed that patients in the high-risk group had higher *TRIB3*, *CAV1*, *SLC7A11*, *AURKA*, and *AKR1C3* expression levels than patients in the low-risk group (Figure 4G). The GEO external validation results were consistent with the TCGA results (Figures 4B,D,F,H).

### Verification of the Expression of the Five FRGs and Prognosis in the GEPIA Online Database

**FIGURE 5:**
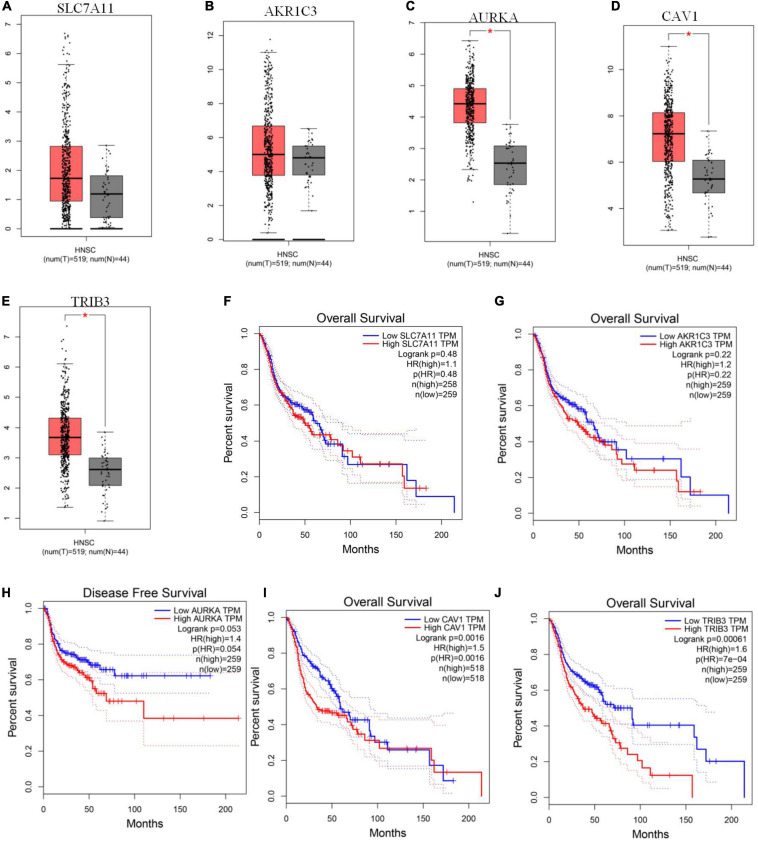
GEPIA verification of ferroptosis-related gene (FRG) expression levels and their relationship with prognosis. **(A–E)** Expression levels of *SLC7A11*, *AKR1C3*, *AURKA*, *CAV1*, and *TRIB3*. **(F–J)** Kaplan-Meier curves for OS or disease-free survival based on the five FRGs. *TRIB3* and *CAV1* are significantly related to OS, *AURKA* is closely related to patient disease-free survival (*P* < 0.05).

We used GEPIA to evaluate the expression levels of the five signature genes in HNSCC and to further determine the accuracy of the FRG-based prognostic model. Consistent with R analysis results, except for *AKR1C3* and *SLC7A11*, expression levels of the remaining three signature genes were significantly higher in HNSCC tissues than in normal tissues (Figures 5A–E). *CAV1* and *TRIB3* expression significantly correlated with OS (Figures 5I,J), while *AURKA* expression was closely related to patient disease-free survival (Figure 5H). There is no significant correlation between the expression of AKR1C3 and the OS of HNSCC patients (Figure 5G). Although there was no significant correlation between *SLC7A11* and prognosis (Figure 5F), a large number of studies have suggested that *SLC7A11* is associated with a poor prognosis. Further research is needed to clarify the relationship between *SLC7A11* and the prognosis of HNSCC patients.

### Calculating the Independent Prognostic Value of the FRG Prognostic Model and Constructing a Nomogram to Evaluate Patient Prognosis

**FIGURE 6:**
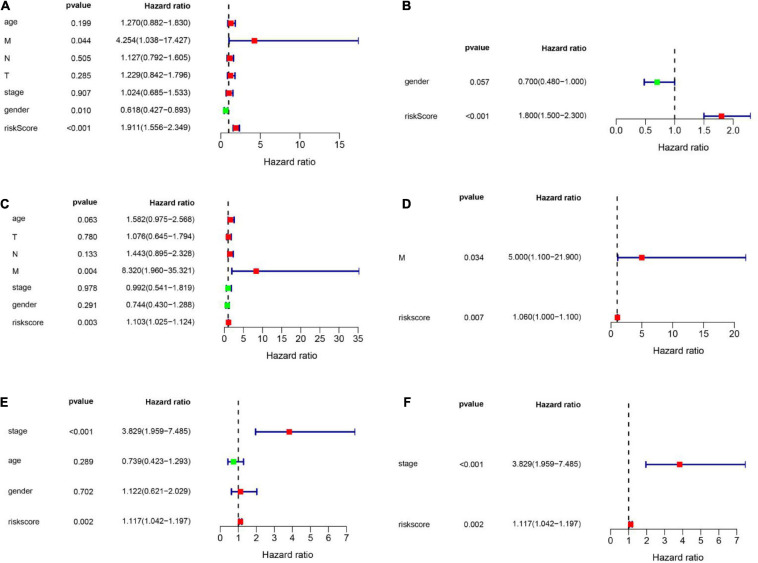
Univariate and multivariate Cox analyses were employed to assess the ability of the risk score and other clinical factors to predict overall survival. The forest plots show the hazard ratio and 95% confidence interval values. **(A,B)** Results of the univariate and multivariate Cox regression analyses regarding OS in the TCGA training set. **(C,D)** Results of the univariate and multivariate Cox regression analyses regarding OS in the TCGA validation set. **(E,F)** Results of the univariate and multivariate Cox regression analyses of OS in the GEO validation set.

**FIGURE 7:**
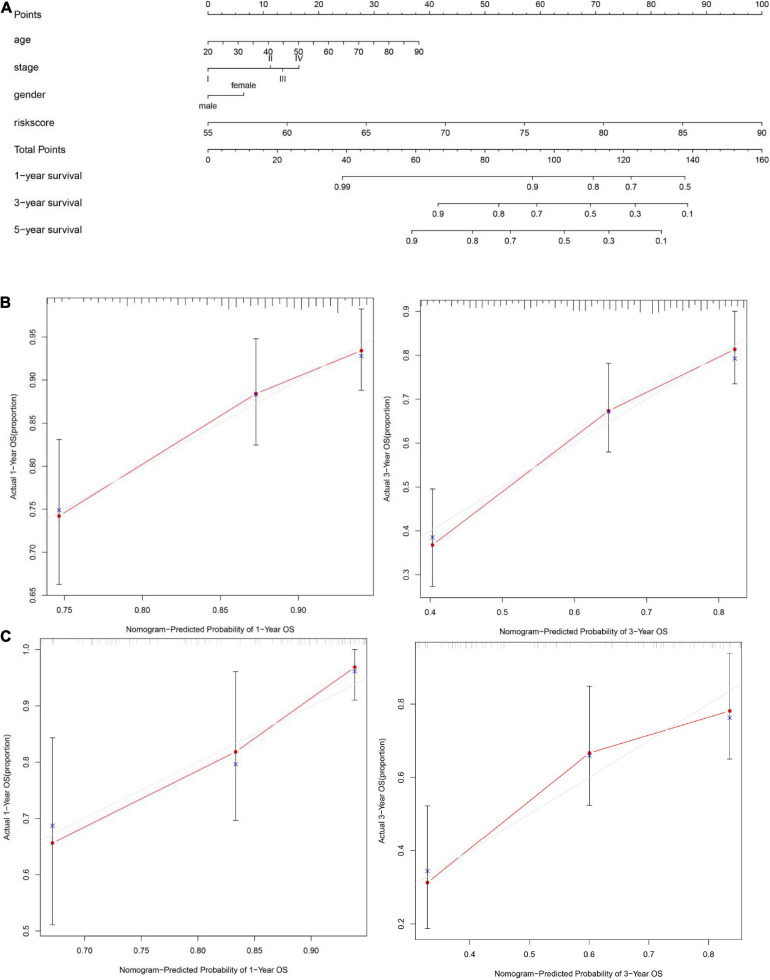
Construction of a nomogram and evaluation patient’s survival rate with HNSCC. **(A)** A nomogram to predict the 1-, 3-, and 5-year overall survival (OS) of patients with HNSCC was constructed with the TCGA training set (*n* = 326). **(B)** A calibration plot of the nomogram was used to predict the 3- and 5-year OS of the cancer genome atlas (TCGA) training set (*n* = 326). **(C)** A calibration plot of the nomogram was used to predict 1- and 3-year OS in the GSE41613 dataset (*n* = 97). The ideal nomogram is represented by the gray line, and the actual nomogram is represented by the red line.

To explore whether the FRG prognostic model is an independent prognostic factor for HNSCC, we used univariate and multivariate Cox regression analyses. Univariate Cox regression analysis revealed that the risk scores in the TCGA training set, TCGA validation set, and GEO validation set were significantly related to OS (TCGA training set: *HR* = 1.911, 95% CI: 1.556–2.349, *P* < 0.001; TCGA validation set: *HR* = 1.103, 95% CI: 1.025–1.124, *P* = 0.003; GSE41613: *HR* = 1.117, 95% CI: 1.042–1.197, *P* = 0.002; Figures 6A,C,E). Multivariate Cox regression analysis was used to adjust for other confounders, and the risk score remained significantly associated with OS (TCGA training set: HR: 1.800, 95% CI: 1.500–2.300, *P* < 0.001; TCGA validation set: HR: 1.060, 95% CI: 1.0–1.1, *P* = 0.007; GEO validation set: HR: 1.117, 95% CI: 1.042–1.197, *P* = 0.002, Figures 6B,D,F). These results confirmed the independent ability of the risk model to predict prognosis in patients with HNSCC (Figure 6). Based on the multivariate Cox analysis, a nomogram was constructed to predict the 1-, 3-, and 5-year OS of patients with HNSCC with the TCGA training set (Figure 7A). The calibration charts for 1- and 3-year OS showed good predictive value in the TCGA training set and the GSE41613 validation set (Figures 7B,C).

### GO and KEGG Enrichment Analyses of the TCGA Training and Validation Sets

**FIGURE 8:**
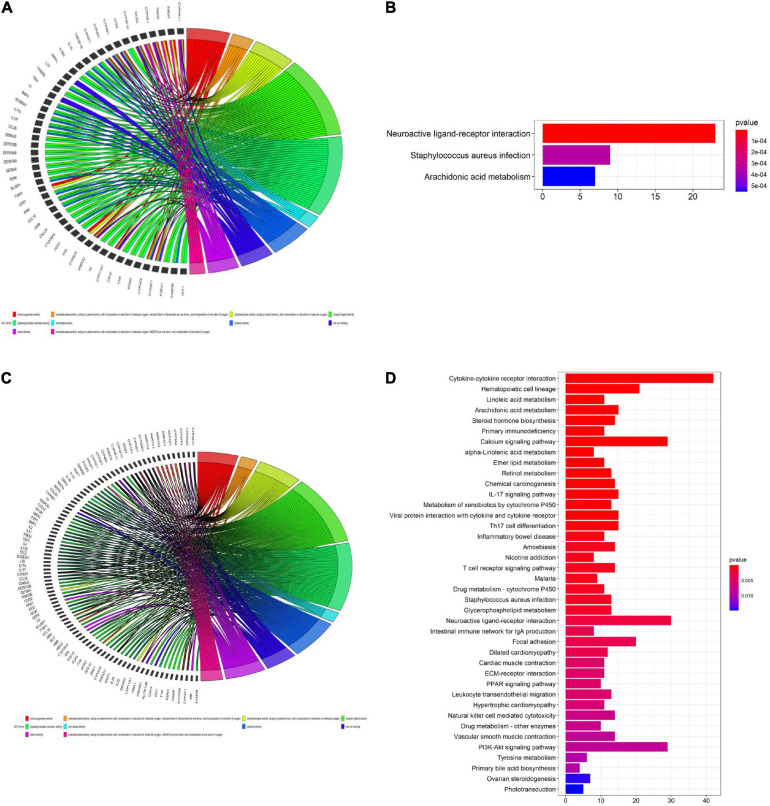
Gene ontology (GO) **(A,C)** and Kyoto encyclopedia of genes and genomes (KEGG) **(B,D)** enrichment analyses. **(A,C)** GO enrichment analysis of the cancer genome atlas (TCGA) training set. **(B)** KEGG enrichment analysis of the TCGA training set. **(C)** GO enrichment analysis of the TCGA validation set. **(D)** KEGG enrichment analysis of the TCGA validation set.

We used the “ClusterProfile” R package to conduct GO and KEGG enrichment analyses of identified DEGs to verify the role of ferroptosis-related pathways in different risk groups. Our results showed that key oxidative stress pathways involved in regulating ferroptosis pathways were enriched in both the TCGA training set and the TCGA validation set (Figures 8A–D). For example, heme binding and iron ion binding were enriched in both the TCGA training set and the TCGA validation set (padj < 0.05, Figures 8A,C). KEGG analysis of the TCGA validation set showed some immune-related pathways were enriched, including pathways related to cytokine-cytokine receptor interactions, IL-17 signaling, and primary immunodeficiency (Figure 8D). GO enrichment analysis of the TCGA training set and TCGA validation set revealed increased cytokine activity (padj < 0.05, Figures 8A,C).

**FIGURE 9:**
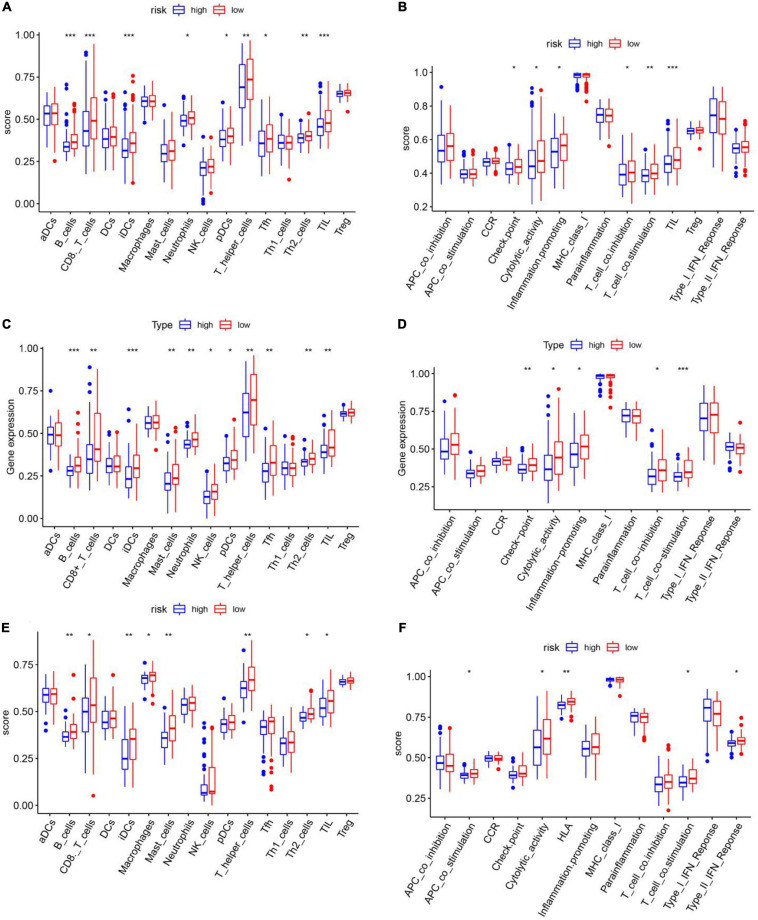
Single-sample gene set enrichment analysis of the immune cell infiltration status of different risk groups in the cancer genome atlas training set. **(A,B)** TCGA validation set **(C,D)** and GEO validation set **(E,F)**. **(A,C,E)** Immune cell infiltration status in high- and low-risk groups. **(B,D,F)** Immune functional enrichment in the different risk groups.

Single-sample gene set enrichment analysis (ssGSEA) was used to calculate the enrichment scores for different immune cell subsets, related functions, and pathways in different risk groups. Surprisingly, in the TCGA training set, B cells, CD8 T cells, induced dendritic cells (iDCs), mast cells, plasmacytoid dendritic cells (pDCs), T follicular helper (Tfh) cells, T helper 1 (Th1) cells, T helper 2 (Th2) cells, and tumor-infiltrating lymphocytes (TILs) were significantly differently enriched between the low-risk and high-risk groups (padj < 0.05, Figures 9A,B). KEGG pathway enrichment analysis revealed that the enrichment of T cell co-stimulation significantly differed between the two risk groups (padj < 0.05, Figure 9B). The terms T cell co-inhibition, inflammation promotion, cytolytic activity, checkpoints, and CCR and APC co-inhibition were obviously enriched in the low-risk group (padj < 0.05, Figure 9B). TCGA validation set and GEO validation set analyses confirmed the differences in B cells, CD8 T cells, iDCs, mast cells, Th2 cells, TILs, cytolytic activity, and T cell co-stimulation between the two risk groups (padj < 0.05, Figures 9C–F).

### Experimental Validation

**FIGURE 10:**
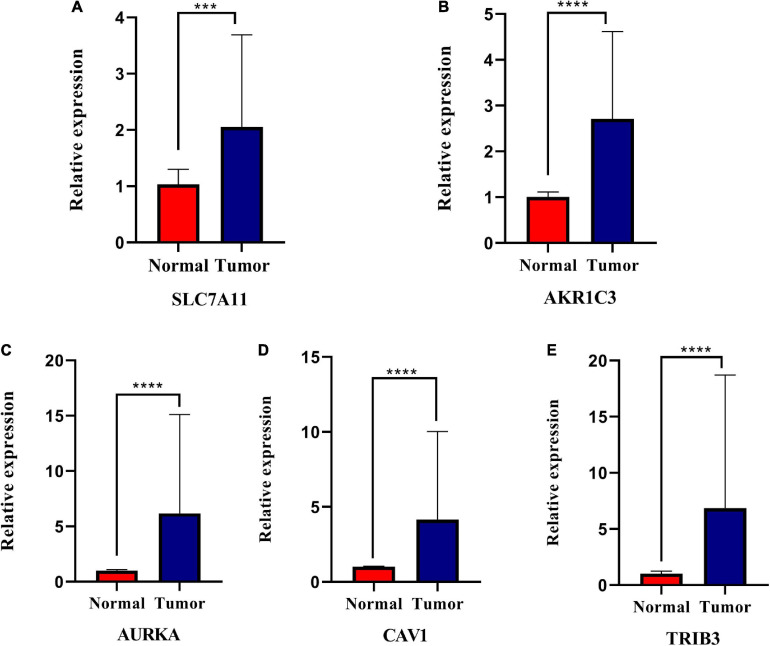
Relative expression levels of the five genes in normal and squamous cell carcinoma of the head and neck tissues (HNSCC). *SLC7A11* **(A)**, *AKR1C3* **(B)**, *AURKA* **(C)**, *CAV1* **(D)**, and *TRIB3* **(E)** are significantly up-regulated in HNSCC tissues. *P < 0.05; **P < 0.01; ***P < 0.001; ****P < 0.0001.

To determine if the expression of these genes significantly differed in clinical tissues, we performed PCR and IHC examinations on clinical specimens. PCR results showed that SLC7A11 (***P < 0.001), CAV1 (****P < 0.0001), AKR1C3 (****P < 0.0001), AURKA (****P < 0.0001) expression was significantly up-regulated in HNSCC tissues (*P* < 0.05, Figures 10A–E and Supplementary Table 5).

**FIGURE 11:**
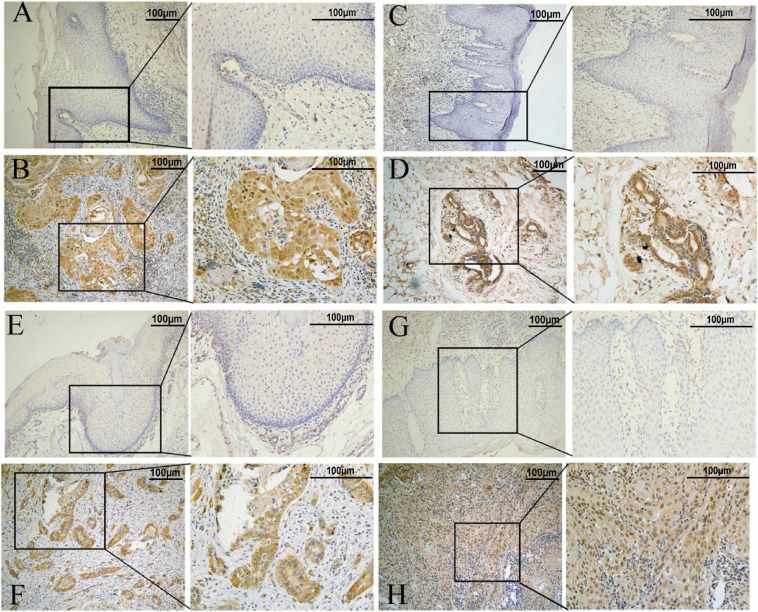
Representative immunohistochemistry results. **(A,C,E,G)** represent normal tissue. **(B,D,F,H)** represents tumor tissue (the scale bar represents 100 μm). **(A,B)** AKR1C3 protein. **(C,D)** AURKA protein. **(E,F)** CAV1protein. **(G,H)** TRIB3 protein.

IHC results showed that the CAV1, AKR1C3, and TRIB3 protein expression levels were significantly higher in HNSCC tumor tissues (*P* < 0.05), but the difference in AURKA was not significant (*P* = 0.144). This may be due to the small sample size (Figures 11A–H and Table 2).

**TABLE 2:** Associations between the clinicopathological characteristics and protein expression in HNSCC.

Characteristic		AKR1C3 expression			CAV1 expression			AURKA expression			TRIB3 expression		
					
		Positive N	Negative N	*p* value	Positive N	Negative N	*p* value	Positive N	Negative N	*p* value	Positive N	Negative N	*p* value
Tissues				0.03			0.001			0.144			0.045
	HNSCC tumors	21	29		31	19		22	28		32	18	
	paired non-tumor tissues	10	40		13	37		14	36		21	29	
**Age(years)**				0.5			0.244			0.612			0.616
	<60	10	15		18	7		11	14		16	9	
	≥60	11	14		13	12		11	14		16	9	
**Gender**				0.543			0.756			0.076			0.757
	Male	13	21		22	12		18	16		21	13	
	Female	8	8		9	7		4	12		11	5	
**T stage**				0.5			0.561			0.154			0.14
	T1-2	11	14		14	11		8	17		13	12	
	T3-4	10	15		17	8		14	11		19	6	
**lymph node metastasis**				0.139			0.569			0.559			0.366
	Yes	5	14		12	7		7	12		14	5	
	No	16	15		19	12		15	16		18	13	
**Metastasis**				0.58			0.62			0.44			0.64
	Yes	0	1		1	0		1	0		1	0	
	No	21	28		30	19		21	28		31	18	
**Stage**				0.232			0.522			0.394			0.035
	I–II	10	8		12	8		7	13		9	11	
	III–IV	11	21		19	11		14	15		23	7	

## Discussion

We systematically calculated the differential expression of FRGs collected from a ferroptosis database in the cancer tissues of patients with HNSCC. We further analyzed the relationship between these differentially expressed FRGs and OS in patients with HNSCC to provide insights into the study of ferroptosis in HNSCC. Ferroptosis is a type of programmed cell death caused by imbalanced iron metabolism, which leads to a reduction in, or the disappearance of, mitochondrial crista and rupture of the mitochondrial outer membrane (Xie et al., 2016; Conrad et al., 2018). Interestingly, ferroptosis has been implicated in several different types of cancer, including lung cancer, melanoma, and hepatocellular carcinoma (Alvarez et al., 2017; Basit et al., 2017; Nie et al., 2018; Ashrafizadeh et al., 2019; Bai et al., 2019; Gai et al., 2020). Recently, an increasing number of basic studies have indicated that ferroptosis caused by iron overload plays an important role in reversing chemoradiotherapy resistance and improving the efficacy of immunotherapy (Sleire et al., 2015; Li et al., 2020). Here, Cox regression analyses were used to construct a ferroptosis-related prognosis model consisting of five genes (*SLC7A11*, *AURKA*, *TRIB3*, *AKR1C3*, and *CAV1*), and internal and external validation was conducted. GO and KEGG enrichment analyses of genes that were differentially expressed in the different risk groups showed significant enrichment in oxidative stress-related pathways associated with ferroptosis, as well as in immune-related pathways. These results are consistent with those of prior work. SLC7A11 is a well-known ferroptosis-regulating factor, and the erastin ferroptosis-inducer targets SLC7A11 to inhibit the cystine glutamate transport system to ultimately inhibit ferroptosis. Recent studies have shown that SLC7A11 expression is significantly lower in HPV-positive tumors than in HNSCC HPV-negative tumors, and that HNSCC with lower SLC7A11 expression is more sensitive to ferroptosis than HNSCC with higher SLC7A11 expression (Hémon et al., 2020). AURKA participates in tumor development by interfering with mitosis and other signaling pathways. AURKA is an important member of the AURK kinase family, which is involved in the occurrence of various tumors and is closely related to tumor prognosis (Bavetsias and Linardopoulos, 2015; Xie et al., 2017). Gomaa et al. (2019) found that *AURKA* was a direct target of miR-4715-3p and that inhibiting *AURKA* or modulating miR-4715-3p could inhibit GPX4 expression, leading to disruption of the intracellular lipid peroxidation clearance barrier and inducing ferroptosis. *TRIB3* is an important stress-related gene that can induce the expression of various stimulating factors and participates in the regulation of transforming growth factor β, phosphatidyl inositol 3 kinase, and SRC (the characteristic activated protein kinase signaling pathways) (Tomcik et al., 2016). The integrated stress response induces ATF4-dependent transcription during periods of negative feedback regulation. ATF4 promotes *TRIB3* expression, and TRIB3 interacts with ATF4 and suppresses *ATF4* transcriptional activity. TRIB3 can bind to Akt and inhibit its activation to block insulin signaling. TRIB3 may directly bind to and mask the “ThR-308” phosphorylation site in AKT1. TRIB3 can also interact with the P65 RELA NF-κB transactivator to inhibit phosphorylation. This inhibits the transcriptional activity of P65 RELA, leading to decreased ATF4 expression and increased erastin-induced cell death. ATF4 cooperates with HSPA5 to prevent GPX4 protein degradation and the induction of ferroptosis (Wu et al., 2003; Bi et al., 2015; Aimé et al., 2020). AKRs are a family of enzymes responsible for inhibiting the cytotoxic potential of aldehydes and ketones by converting them into corresponding alcohols. Thus, ferroptosis resistance may be related to the detoxification activity of AKRs, as it leads to a reduction in lipid peroxides, which are key operators of the ferroptosis process (Gagliardi et al., 2019). CAV1 protects hepatocytes from ferroptosis in autoimmune hepatitis (Deng et al., 2020). With the exception of *SLC7A11*, the mechanisms of the genes in ferroptosis are not clearly known. Therefore, whether these genes regulate ferroptosis and control the prognosis of patients with HNSCC is unclear. In 2019, Professor Wang et al. (2019) proposed, for the first time, that CD8 T cells partially induce tumor cell death by regulating ferroptosis (Wang et al., 2019), but the specific mechanism involved remains unclear. In this study, we conducted GO and KEGG enrichment analyses of the DEGs in the different risk groups and found that many immune cells and immune functions were enriched. This result confirms that ferroptosis is associated with immunity. Previous studies have suggested that CD8^+^ T cells can secrete IFNγ, promote the production of intracellular lipid peroxides, induce ferroptosis, and increase the efficacy of tumor immunotherapy. In this study, we found that the number of CD8^+^T cells were significantly higher in the low-risk group than in the high-risk group, which is consistent with the present study. B cells have a variety of immune response functions. Tumor-infiltrating B lymphocytes can promote the T cell response and directly kill tumor cells by secreting immunoglobulins, thereby inhibiting tumor progression. B cells were significantly enriched in the low-risk group. These results indicate that B cells may also mediate ferroptosis to inhibit tumor progression. The number of iDCs was also significantly higher in the low-risk group. iDCs demonstrate effective antigen presentation following stimulation by cytotoxic T lymphocytes, thereby activating the immune response, which may also be related to ferroptosis. However, it is undeniable that these results still need further in-depth research for confirmation.

There are several limitations to this study. First, we built a prognostic model based on the TCGA public database and verified it with the GEO database. The number of samples was limited, and most of the samples were from European and American white populations. There was a paucity of samples from Asians or other races. More data are needed to verify the clinical applicability of the risk model. Second, the inherent weakness of only considering factors related to ferroptosis to establish a prognostic model is inevitable because many significant prognostic-related genes in HNSCC may have been excluded. In addition, it is worth noting that the correlation between the ferroptosis risk score and immunity has not been verified at the cellular level.

In summary, our study constructed a prognostic model based on five FRGs. This model was proven to be independently related to OS in both the training set and the validation set, providing a new molecular target for the prediction of HNSCC prognosis. The mechanism underlying the relationship between the FRGs and the infiltration status of immune cells in HNSCC is still unclear, and further research is needed.

## Data Availability Statement

The original contributions presented in the study are included in the article/Supplementary Material, further inquiries can be directed to the corresponding author/s.

## Ethics Statement

The study was approved by the Internal Review Committee of The Third Affiliated Hospital of Kunming Medical University. The patients/participants provided their written informed consent to participate in this study.

## Author Contributions

CL and XW: study design, drafting the manuscript, interpretation of data, and statistical processing. CL, XW, RQ, and ZZ: acquisition and analysis of data. CS: reviewing and approving the final version of the manuscript. All authors read and approved the final manuscript.

## Conflict of Interest

The authors declare that the research was conducted in the absence of any commercial or financial relationships that could be construed as a potential conflict of interest.

## Publisher’s Note

All claims expressed in this article are solely those of the authors and do not necessarily represent those of their affiliated organizations, or those of the publisher, the editors and the reviewers. Any product that may be evaluated in this article, or claim that may be made by its manufacturer, is not guaranteed or endorsed by the publisher.

## Footnotes

1
https://asia.ensembl.org/index.html/

2
https://www.ncbi.nlm.nih.gov/geo/

3
http://www.zhounan.org/ferrdb

4
http://bioinformatics.psb.ugent.be/webtools/Venn/

5
http://gepia.cancer-pku.cn/
