# Supplementary material for: Identification of a Ferroptosis Gene Set That Mediates the Prognosis of Squamous Cell Carcinoma of the Head and Neck
Source: Front Genet. 2021 Sep 3;12:698040. doi: 10.3389/fgene.2021.698040 (PMC8446460; doi:10.3389/fgene.2021.698040)
Supplement: Supplementary file 5 [file Table_5.DOC]

| IHC detection of protein expression level(HNSCC tumor tissues) | | | | | | | | | | |
| --- | --- | --- | --- | --- | --- | --- | --- | --- | --- | --- |
| number | age | M | N | T | stage | gender | AKR1C3 | AURKA | CAV1 | TRIB3 |
| 1 | 51 | M0 | N2b | T3 | IVA | male | - | - | - | +++ |
| 2 | 53 | M0 | N0 | T3 | III | male | ++ | ++ | ++ | - |
| 3 | 64 | M0 | N0 | T4a | IVA | male | - | + | +++ | ++ |
| 4 | 77 | M0 | N0 | T2 | II | male | ++ | - | - | - |
| 5 | 55 | M0 | N0 | T3 | III | male | - | + | ++ | +++ |
| 6 | 57 | M0 | N2 | T2 | IVA | female | - | - | + | - |
| 7 | 59 | M0 | N0 | T3 | III | female | +++ | ++ | - | + |
| 8 | 60 | M0 | N2c | T4a | IVA | female | - | - | ++ | ++ |
| 9 | 58 | M0 | N2b | T2 | IVA | male | ++ | - | - | + |
| 10 | 72 | M0 | N0 | T4a | IVA | male | - | ++ | +++ | +++ |
| 11 | 76 | M0 | N0 | T2 | II | male | - | + | - | ++ |
| 12 | 53 | M0 | N2b | T3 | IVA | male | ++ | - | ++ | + |
| 13 | 88 | M0 | N0 | T1 | I | male | - | ++ | - | - |
| 14 | 33 | M0 | N0 | T2 | II | male | ++ | - | ++ | + |
| 15 | 52 | M0 | N0 | T4a | IVA | male | - | + | + | ++ |
| 16 | 25 | M0 | N1 | T3 | III | female | - | - | ++ | ++ |
| 17 | 66 | M0 | N0 | T4b | IVB | male | + | + | - | + |
| 18 | 41 | M0 | N0 | T3 | III | male | +++ | - | ++ | ++ |
| 19 | 48 | M0 | N0 | T3 | III | male | - | +++ | + | - |
| 20 | 58 | M0 | N1 | T3 | III | male | - | ++ | + | - |
| 21 | 81 | M0 | N2c | T4 | IVA | male | ++ | ++ | - | +++ |
| 22 | 59 | M0 | N0 | T2 | II | male | + | - | + | - |
| 23 | 67 | M0 | N0 | T3 | III | male | + | - | - | + |
| 24 | 55 | M0 | N0 | T2 | II | male | - | + | ++ | ++ |
| 25 | 54 | M0 | N0 | T2 | II | male | - | - | + | + |
| 26 | 67 | M0 | N0 | T2 | II | female | - | - | - | - |
| 27 | 46 | M0 | N1 | T1 | III | female | - | - | - | + |
| 28 | 82 | M0 | N2c | T4a | IVA | female | + | - | + | - |
| 29 | 65 | M0 | N0 | T4a | IVA | female | + | - | - | + |
| 30 | 77 | M1 | N1 | T1 | IVC | female | - | ++ | +++ | +++ |
| 31 | 41 | M0 | N2b | T2 | IVA | male | - | - | - | + |
| 32 | 53 | M0 | N1 | T4a | IVA | male | - | - | ++ | ++ |
| 33 | 77 | M0 | N2 | T3 | IVA | male | - | + | - | ++ |
| 34 | 79 | M0 | N2 | T3 | IVA | female | + | - | ++ | + |
| 35 | 74 | M0 | N1 | T4a | IVA | male | - | ++ | + | - |
| 36 | 63 | M0 | N2b | T4a | IVA | male | - | - | + | + |
| 37 | 45 | M0 | N1 | T3 | III | male | - | + | + | - |
| 38 | 46 | M0 | N1 | T3 | III | male | - | + | - | ++ |
| 39 | 58 | M0 | N0 | T2 | II | male | + | ++ | + | - |
| 40 | 62 | M0 | N0 | T1 | I | male | - | - | + | + |
| 41 | 78 | M0 | N0 | T2 | II | female | - | - | + | + |
| 42 | 42 | M0 | N0 | T1 | I | male | + | - | + | - |
| 43 | 84 | M0 | N0 | T2 | II | female | +++ | - | - | ++ |
| 44 | 65 | M0 | N0 | T2 | II | female | + | - | + | - |
| 45 | 71 | M0 | N0 | T2 | II | male | - | + | + | - |
| 46 | 52 | M0 | N0 | T2 | II | female | - | + | - | + |
| 47 | 78 | M0 | N0 | T1 | I | male | - | - | - | - |
| 48 | 62 | M0 | N0 | T1 | I | female | ++ | + | - | - |
| 49 | 59 | M0 | N0 | T2 | II | male | + | - | + | - |
| 50 | 88 | M0 | N0 | T2 | II | female | + | - | + | + |

| IHC detection of protein expression level(paired non-tumor tissues) | | | | | | | | | | |
| --- | --- | --- | --- | --- | --- | --- | --- | --- | --- | --- |
| number | age | M | N | T | stage | gender | AKR1C3 | AURKA | CAV1 | TRIB3 |
| 1 | age | M0 | N2b | T3 | IVA | male | - | ++ | - | ++ |
| 2 | 51 | M0 | N0 | T3 | III | male | ++ | - | ++ | - |
| 3 | 53 | M0 | N0 | T4a | IVA | male | - | - | ++ | - |
| 4 | 64 | M0 | N0 | T2 | II | male | - | ++ | - | + |
| 5 | 77 | M0 | N0 | T3 | III | male | - | - | - | + |
| 6 | 55 | M0 | N2 | T2 | IVA | female | - | - | + | - |
| 7 | 57 | M0 | N0 | T3 | III | female | - | + | - | ++ |
| 8 | 59 | M0 | N2c | T4a | IVA | female | - | + | - | - |
| 9 | 60 | M0 | N2b | T2 | IVA | male | - | - | ++ | - |
| 10 | 58 | M0 | N0 | T4a | IVA | male | - | - | - | - |
| 11 | 72 | M0 | N0 | T2 | II | male | - | - | - | - |
| 12 | 76 | M0 | N2b | T3 | IVA | male | + | ++ | - | +++ |
| 13 | 53 | M0 | N0 | T1 | I | male | - | - | - | + |
| 14 | 88 | M0 | N0 | T2 | II | male | ++ | - | - | - |
| 15 | 33 | M0 | N0 | T4a | IVA | male | - | - | + | - |
| 16 | 52 | M0 | N1 | T3 | III | female | - | ++ | - | + |
| 17 | 25 | M0 | N0 | T4b | IVB | male | + | - | - | ++ |
| 18 | 66 | M0 | N0 | T3 | III | male | - | + | - | - |
| 19 | 41 | M0 | N0 | T3 | III | male | - | - | - | + |
| 20 | 48 | M0 | N1 | T3 | III | male | - | +++ | - | + |
| 21 | 58 | M0 | N2c | T4 | IVA | male | ++ | - | - | +++ |
| 22 | 81 | M0 | N0 | T2 | II | male | - | - | + | - |
| 23 | 59 | M0 | N0 | T3 | III | male | - | - | - | + |
| 24 | 67 | M0 | N0 | T2 | II | male | - | - | - |  |
| 25 | 55 | M0 | N0 | T2 | II | male | - | - | - | + |
| 26 | 54 | M0 | N0 | T2 | II | female | - | - | - | - |
| 27 | 67 | M0 | N1 | T1 | III | female | - | ++ | + | - |
| 28 | 46 | M0 | N2c | T4a | IVA | female | - | - | - | - |
| 29 | 82 | M0 | N0 | T4a | IVA | female | + | - | - | + |
| 30 | 65 | M1 | N1 | T1 | IVC | female | - | +++ | - | - |
| 31 | 77 | M0 | N2b | T2 | IVA | male | - | - | - | + |
| 32 | 41 | M0 | N1 | T4a | IVA | male | - | - | ++ | - |
| 33 | 53 | M0 | N2 | T3 | IVA | male | - | - | - | - |
| 34 | 77 | M0 | N2 | T3 | IVA | female | + | - | +++ | - |
| 35 | 79 | M0 | N1 | T4a | IVA | male | - | + | - |  |
| 36 | 74 | M0 | N2b | T4a | IVA | male | - | - | - | ++ |
| 37 | 63 | M0 | N1 | T3 | III | male | - | - | + | - |
| 38 | 45 | M0 | N1 | T3 | III | male | - | + | - | - |
| 39 | 46 | M0 | N0 | T2 | II | male | + | - | - | + |
| 40 | 58 | M0 | N0 | T1 | I | male | - | - | - | - |
| 41 | 62 | M0 | N0 | T2 | II | female | - | - | - | - |
| 42 | 78 | M0 | N0 | T1 | I | male | - | - | + | + |
| 43 | 42 | M0 | N0 | T2 | II | female | +++ | - | - | - |
| 44 | 84 | M0 | N0 | T2 | II | female | - | - | ++ | - |
| 45 | 65 | M0 | N0 | T2 | II | male | - | - | - | - |
| 46 | 71 | M0 | N0 | T2 | II | female | - | + | - | - |
| 47 | 52 | M0 | N0 | T1 | I | male | - | - | - | - |
| 48 | 78 | M0 | N0 | T1 | I | female | ++ | ++ | - | + |
| 49 | 62 | M0 | N0 | T2 | II | male | - | - | ++ | - |
| 50 | 59 | M0 | N0 | T2 | II | female | - | - | - | - |

| qRT-PCR detection mRNA level | | | | | | |
| --- | --- | --- | --- | --- | --- | --- |
| number | age | M | N | T | stage | gender |
| 1 | 65 | M0 | N0 | T2 | II | male |
| 2 | 62 | M0 | N0 | T2 | II | male |
| 3 | 77 | M0 | N2 | T3 | IVA | female |
| 4 | 41 | M0 | N1 | T4a | IVA | male |
| 5 | 77 | M0 | N0 | T3 | III | male |
| 6 | 67 | M0 | N1 | T1 | III | female |
| 7 | 57 | M0 | N0 | T3 | III | female |
| 8 | 60 | M0 | N2b | T2 | IVA | male |
| 9 | 60 | M0 | N2b | T2 | IVA | male |
| 10 | 58 | M0 | N0 | T4a | IVA | male |
